# Nutraceutical Combinations in Hypercholesterolemia: Evidence from Randomized, Placebo-Controlled Clinical Trials

**DOI:** 10.3390/nu13093128

**Published:** 2021-09-08

**Authors:** Olga Protic, Anna Rita Bonfigli, Roberto Antonicelli

**Affiliations:** 1 Cardiology Unit, IRCCS INRCA, 60129 Ancona, Italy; r.antonicelli@inrca.it; 2 Scientific Direction, IRCCS INRCA, 60129 Ancona, Italy; a.bonfigli@inrca.it

**Keywords:** hypercholesterolemia, LDL-cholesterol, dietary supplements, nutraceuticals, combinations, primary prevention, randomized clinical trials, red yeast rice, cardiovascular disease, prevention, vitamins, coenzyme Q, policosanols, phytosterols

## Abstract

There is an increasing number of nutraceutical combinations (NCs) on the market for hypercholesterolemia, although clinical trials to verify their safety and efficacy are scarce. We selected fourteen randomized, placebo-controlled clinical trials (RCTs) on different lipid-lowering NCs in hypercholesterolemic subjects. We described each compound′s mechanism of action and efficacy in the mixtures and summarized the clinical trials settings and NCs safety and efficacy results. Almost all NCs resulted efficient against hypercholesterolemia; only one reported no changes. Interestingly, red yeast rice (RYR) was present in eleven mixtures. It is not clear whether the lipid-lowering efficacy of these combinations derives mainly from the RYR component monacolin K “natural statin” single effect. Up to now, few RCTs have verified the efficacy of every single compound vs. NCs to evaluate possible additive or synergistic effects, probably due to the complexity and the high resources request. In conclusion, to manage the arising nutraceutical tide against hypercholesterolemia, it could be helpful to increase the number and robustness of clinical studies to verify the efficacy and safety of the new NCs.

## 1. Introduction

Cardiovascular diseases (CVDs) remain the leading cause of death worldwide [1]. Atherosclerotic plaque formation is an inflammatory process in the endothelial vessel wall associated with retained low-density lipoprotein (LDL) [2,3]. Elevated plasma cholesterol levels (hypercholesterolemia) are a major cardiovascular risk factor. Strategies to combat elevated plasma LDL cholesterol include lifestyle changes and pharmacological interventions [4,5].

Despite an incontrovertible efficacy of lipid-lowering therapy in reducing the incidence of cardiovascular events, a significant number of people refuse drug therapy. Nutraceuticals have the potential as an alternative approach and treatment for hypercholesterolemia. Moreover, the European Society of Cardiology and the European Atherosclerosis Society guidelines, as well as the International Lipid Expert Panel, recommend using dietary supplements and a balanced diet to improve lipid profile [6,7].

Nutraceuticals are bioactive compounds of plant or microbial origin, with possible beneficial effects on human health. Some nutraceuticals are prescribed as lipid-lowering substances. Several meta-analyses highlighted the efficacy of common nutraceuticals with a different mechanism of action (for example, fiber, phytosterols, red yeast rice, berberine) to treat hypercholesterolemia in maintaining the physiological levels of plasma cholesterol [8,9]. It is less known about the efficacy and safety of their combinations while, as frequently occurs for many nutraceuticals, their offer in the marketplace is growing for commercial interest. Starting from these observations, in this review, we evaluated the most robust clinical evidence on the use and efficacy of lipid-lowering NCs in the form of capsules in hypercholesterolemic subjects. We described the mechanism of action of each compound in the mixtures, summarized the clinical trials settings and the NCs safety and efficacy.

## 2. Methodology

We searched the MEDLINE (PubMed) to identify eligible articles published up to the end of May 2021. The articles investigating the effects of nutraceutical combinations on the hypercholesterolemic populations analyzed by placebo-controlled RCTs were considered. RCTs are the “gold standard” of clinical trials investigating the relationships between an intervention and a health outcome. The following keywords were used: hypercholesterolemia, nutraceutical combinations, dietary supplements, and filtered by a randomized clinical trial. The total articles found manually excluded those that did not correspond to the topic of interest. To cover information of NC effects on the general population, we have excluded: RCTs that observed only particular populations such as subjects with age under 18 or over 75, populations with specific conditions such as hypertension, familial hypercholesterolemia, postmenopausal women, subjects in secondary prevention with history of CVDs. When multiple RCTs for a specific NC were found, we included RCT with more subjects included in the study. The selected nutraceutical combinations are presented in Table 1.

## 3. Lipid-Lowering Molecular Mechanism of Action and Efficacy of Single Compound Present in the NC

*Red yeast rice* (*Monascus purpureus*). The extract of red yeast rice (RYR) containing the active ingredient monacolin K has the most cholesterol-lowering potential on the market. It is reported that in humans, the use of 3 to 10 mg daily of monacolin K reduces LDL cholesterol plasma levels between 15% and 25% after 6 to 8 weeks [24]. RYR consists of 25% to 73% sugars, 14% to 31% proteins, 2% to 7% water, 1% to 5% fatty acids, sterols, isoflavones, pigments, and polyketides [25]. Substances produced by the fermentation of yeast and rice, Monacolin K, is responsible for its cholesterol-lowering effect on the same statin-like mechanism of action [26]. Monacolin K has the same chemical structure as synthetic lovastatin, and it inhibits hydroxy-3-methylglutaryl CoA (HMG-CoA) reductase and thus cholesterol synthesis.

Gerards et al., in a recent meta-analysis, included 20 clinical trials studies to evaluate the efficacy and safety of RYR. The results demonstrated that doses from 1200 mg/day to 4800 mg/day of RYR (4.8 mg to 24 mg of Monacolin K) reduced LDL-cholesterol on average by 1.02 mmol/L (range, −1.20 to −0.83; 39.4 mg/dL) compared with placebo after 2–24 months of its use. These results were similar to the effects of 40 mg of pravastatin, 20 mg of lovastatin, and 10 mg of simvastatin [6,27].

*Vitamins.* It is known that elevated plasma total homocysteine (Hcy) has been associated with cardiovascular risk and is considered a risk factor for atherosclerosis. B9 vitamin (folate) leads to coronary plaque regression by lowering Hcy [28]. A mild folate deficiency induces a proatherosclerotic phenotype in endothelial cells [29]. Moreover, deficiency in vitamin B12 induces cholesterol biosynthesis by modulating the methylation of Sterol Regulatory Element Binding Transcription Factor 1 (SREBF1) and LDL receptor genes in human adipocytes [30]. The mechanism of vitamin B3 (niacin) action on the lipid profile is well described [31]. It is known that niacin, a metabolite converted into ubiquitous and essential NAD(P) cofactor [32], increases high-density lipoprotein (HDL) cholesterol by inhibiting the selective uptake and degradation of apolipoprotein A, the carrier of HDL particles, in the liver [33]. In this way, the carrier protein is more available for binding to HDL and prolongs its half-life. It has been demonstrated in animal models that vitamin B2 deficiency affects lipid metabolism by reducing apolipoprotein B100 synthesis [34]. Vitamin E is an antioxidant and an essential factor against oxidative stress-related diseases [35]. It is well known that atherosclerosis is the result of hyperlipidemia and lipid oxidation. Thus, this vitamin, with its properties, could prevent lipid oxidation. Many preclinical studies have shown a preventive effect of vitamin E in plaque formation, but clinical data have not supported these findings [36].

Satapathy et al., in a recent multi-arm clinical trial, investigated the effects of folic acid and vitamin B12 on glycaemic control, insulin resistance, and serum lipid profile in subjects with type 2 diabetes mellitus. The significant effect of B12 in plasma insulin, insulin resistance, and serum adiponectin have been demonstrated. However, the improvement in the lipid profile was not found [37]. A clinical study researched the effect of nicotinamide in managing hyperphosphatemia in pediatric patients on regular hemodialysis. After 6 months of treatment with 100 mg twice or three times daily, the mean high-density serum cholesterol levels were increased from 36.2 ± 6.9 mg/dl at baseline to 39.8 ± 3.8 mg/dl (*p* = 0.01) [38]. Ajuluchukwu et al., in a clinical study, showed that patients who were treated with vitamin E daily for 4 weeks showed less than 5% effects on LDL cholesterol [39]. However, the importance of its role on endothelial function and arterial stiffness has been shown by RCTs [40]. The single lipid-lowering effects of vitamin B2 and vitamin B6 and its molecular mechanism of action still need to be researched in humans.

*Coenzyme Q10* (CoQ10). Atherosclerosis is considered a chronic inflammatory disease [41]. Oxidative stress induced by reactive oxygen species (ROS) is a critical mechanism in the atherosclerosis process. CoQ10 acts as an essential cofactor for ATP production, plays a role in mitochondrial bioenergy, and has important antioxidant activities in the body [42]. Moreover, it inhibits LDL oxidation. It also decreases pro-inflammatory cytokines and decreases blood viscosity [43]. Thus, having positive effects on inflammation grade and low oxidative stress could also prevent atherosclerosis process formation and its complications. Moreover, ubiquinol, the reduced form of coenzyme Q10, significantly ameliorated dyslipidemia-related endothelial dysfunction [44]. The antioxidative potential of CoQ10 is very similar to that of vitamin E. Still, the difference is that the source of vitamin E depends exclusively on the diet and hepatic reserves, with no endogenous synthesis as with CoQ10.

Jorat et al., in a meta-analysis that included eight clinical trials, researched the effects of coenzyme Q10 supplementation on lipid profiles among patients with coronary artery diseases. The results showed that CoQ10 significantly decreased total-cholesterol (standard mean differences (SMD): −1.07, 95% confidence interval (CI): −1.94, −0.21, *p* = 0.01). However, significant effects of CoQ10 on LDL cholesterol were not found [45].

*Berberine* (*Berberis* species). Berberine (BBR) is an alkaloid drug extracted from plants that belong to the *Berberis* species. There are several molecular cholesterol-lowering mechanisms of action of BBR. It has been demonstrated on HepG2 human hepatoma cells that its extracts can inhibit cholesterol synthesis by activating AMP kinase [46]. In human hepatoma cell line HepG2 and HEK-293, BBR increased the expression of the LDL receptor (LDLR) and its half-life via the JNK/c-jun pathway and stabilized its mRNA by extracellular signal-regulated kinase (ERK) modulation [22,47]. Considering that proprotein convertase subtilisin/kexin type 9 (PCSK9) mediates LDLR lysosomal degradation in HepG2 cells and that BBR inhibits PCSK9, it is possible that in this way, it should also prolong LDL clearance [48].

Dong et al., in a meta-analysis that included eleven clinical trial studies, showed that the administration of berberine produced a significant reduction in total cholesterol (mean difference −0.61 mmol/L; 95% CI: −0.83 to −0.39), and LDL cholesterol levels (mean difference −0.65 mmol/L; 95% CI: −0.76 to −0.54) [49].

*Policosanols*. Policosanols have an inhibitory effect on HMG-CoA reductase and bile acids absorption. Policosanols inhibit cholesterol synthesis in hepatoma cells by activation of AMP-kinase [50]. Moreover, policosanols modulate HMG-CoA reductase activity in cultured fibroblasts [51].

Berthold et al. researched the effects of policosanols on lipid levels. They divided 143 participants into groups treated with 10, 20, 40, and 80 mg of policosanols or placebo. Nobody in the groups showed a >10% LDL cholesterol reduction after 12 weeks [52]. Another clinical trial confirmed that policosanols did not significantly change the lipid profile of hypercholesterolemic participants [53]. However, other beneficial effects of policosanols on cardiovascular diseases have been researched in different RCTs [54].

*Minerals.* A correlation between abnormality in serum calcium and phosphorous metabolism on lipid profile has been shown, even though the underlying molecular mechanisms are still unknown [55]. There is no evidence of a direct relationship between mineral status and atherosclerosis in humans. The single lipid-lowering effects of minerals still need to be researched in human studies. 

*Phytosterols.* Phytosterols and their esterified derivates, stanols, are structurally similar to cholesterol. They are poorly absorbed in the gut [24,56]. The mechanism of action of the phytosterols is through the inhibition of intestinal absorption of cholesterol. Plant sterols compete with the cholesterol for incorporation into micelles, thereby reducing cholesterol entrance into enterocytes and chylomicrons. The difference to the cholesterol incorporation is that the plant sterols content is carried back into the intestinal lumen by the adenosine 5′-triphosphate (ATP)-binding cassette transporters G5 and G8 transporters (ABCG5/ABCG8) and excreted in the feces [57]. 

Talati et al., in a meta-analysis that included 14 clinical studies, highlighted that in doses up to 3 g/day, there were no differences in efficacy on cholesterolemia between stanols and sterols [58]. Moreover, a meta-analysis with 124 studies found that phytosterols intakes of 0.6–3.3 g/day gradually reduce LDL cholesterol concentrations by, on average, 6–12% [59]. 

*Olive* (*Olea europaea*). The beneficial effects of hydroxytyrosol (HTyr) and tyrosol phenolic antioxidants from Olea Europea have been well described on human health and atherosclerosis [60]. LDL cholesterol oxidation is the key step in the initial process of atherosclerosis. HTyr has the potential to prevent LDL oxidation [61]. HTyr has a potent antioxidant activity as a free radical-scavenger and metal-chelator [62].

Lockyer et al. demonstrated that the use of phenolic-rich olive leaf extract containing 136 mg of oleuropein and 6 mg of hydroxytyrosol reduces total plasma cholesterol (−0.32 (±SD 0.70) mmol/L, *p* = 0.002) and LDL cholesterol (−0.19 (±SD 0.56) mmol/L, *p* = 0.017) [63].

*Artichoke leaf extract* (Cynara *scolymus*). Active ingredients of artichoke that have previously shown a lipid-lowering effect are polyphenols, phytosterols, and fibers [64]. The impact of artichoke leaf extract on lipid profile occurs mainly through luteolin and chlorogenic acid on the cholesterol synthesis process [65,66]. Luteolin decreases hepatocyte nuclear factor 4α (HNF4α) expression. Reduced HNF4α expression also reduced the level of very-low-density lipoprotein VLDL and LDL through decreasing apolipoprotein B [65]. Moreover, luteolin decreases cholesterol synthesis due to inhibition of sterol-regulatory element-binding protein (SREBP-2) and 3-hydroxy-3-methylglutaryl-CoA reductase (HMGCR) [67]. On the other hand, chlorogenic acid decreases cholesterol synthesis due to the inhibition of SREBP-2 through stimulation of AMP-activated protein kinase [68].

A meta-analysis from Sahebkar et al. of data from 9 trials that researched the lipid-lowering effect of artichoke showed a significant decrease in plasma concentrations of total cholesterol (WMD: −17.6 mg/dL, 95% CI: −22.0, −13.3, *p* < 0.001), and LDL cholesterol (WMD: −14.9 mg/dL, 95% CI: −20.4, −9.5, *p* = 0.011) [69].

*Garlic* (*Allium sativum).* Biological lipid-lowering mechanisms of action of garlic oil are similar to that of statins. It inhibits cholesterol synthesis by deactivating HMG-CoA reductase through enhanced phosphorylation. Still, it does not change the levels of mRNA of this enzyme in cultured rat hepatocytes [70]. Moreover, it has been shown that synthetic diallyl disulfide analogs significantly decrease in 3-hydroxy-3-methylglutaryl-CoA reductase (HMGR) activity in hypercholesterolemic rats [71].

A clinical study on the lipid-lowering effects of time-released garlic powder tablets-Allicor of 600 mg daily, in 42 men with mild hypercholesterolemia, showed a reduction in total cholesterol by 7.6% and LDL cholesterol by 11.8% after 12 weeks of treatment [72]. Moreover, a meta-analysis with 39 primary trials that analyzed the effect of garlic on serum lipids showed that garlic used for longer than two months effectively reduces the total serum cholesterol by 17 ± 6 mg/dL and LDL cholesterol by 9 ± 6 mg/dL [73].

*Astaxanthin.* The carotenoid astaxanthin has antioxidant activity and anti-inflammatory properties [74]. There is much evidence suggesting that astaxanthin could exert preventive actions against atherosclerosis targeting different steps in its processes such as oxidative stress, inflammation, and lipid metabolism [75].

A meta-analysis of 7 studies that analyzed the lipid profile and glucose changes after supplementation with astaxanthin did not show any significant effect of astaxanthin on plasma concentrations of total cholesterol [76].

*Banaba* (*Lagerstroemia speciose*). Banaba has anti-diabetic and anti-obesity properties [77]. The exact mechanism of action still needs to be revealed. Several processes have been considered to have beneficial effects on lipid and glucose metabolism: enhance cellular uptake of glucose, decrease gluconeogenesis, and regulate lipid metabolism. It is proposed that these processes could be mediated by peroxisome proliferator-activated receptor, mitogen-activated protein kinase, nuclear factor kappa-B, and other transduction signal factors [78]. On the other hand, the antioxidative effect of banaba has also been studied. The banaba leaf inhibits lipid peroxidation and neutralizes reactive oxygen species such as hydrogen peroxide, nitric oxide, and superoxide [79], contributing to the prevention of arteriosclerosis place formation.

The single effect of *lagerstroemia speciosa* efficacy on lipid profile still needs to be researched by RCT. However, the efficacy of the hypoglycemic effects of banaba is well known. It plays a relevant role in CVD risk prevention [80].

*Curcumin* (*Curcuma longa*). Curcumin is a polyphenol compound found in *Curcuma longa*. Preclinical studies have shown that curcumin inhibits cholesterol biosynthesis via transcriptional inhibition of HMG-CoA reductase, independently from acyl-CoA/cholesterol acetyl transferases that regulate the synthesis of cholesteryl esters [81]. Curcumin attenuates lipogenesis in the liver and inflammatory pathway in adipocytes, preventing high-fat diet-induced insulin resistance and obesity in animal models [82]. Moreover, curcumin stimulates bile acids secretion and decreases the accumulation of lipids in adipose tissue [83,84,85].

Sahebkar et al., in a meta-analysis with five clinical trial studies, investigated the effects of curcumin on blood lipid levels and showed no significant changes [86]. However, another clinical study showed that a curcumin extract capsule of 630 mg taken thrice daily for 12 weeks reduces LDL cholesterol (from 121 ± 37 to 107 ± 25 mg/dL, *p* < 0.05) [87].

*Guggul* (*Commiphora Mukul)*. Guggul is a resin from *Commiphora Mukul* tree. It has been demonstrated that E- and Z-guggulsterones were antagonists of the farnesoid X receptor (FXR) [88]. FXR is a nuclear hormone receptor that controls the regulation of the cholesterol-7-alpha-hydroxylase (CYP7A) gene. In general, CYP7A mediates bile acids synthesis in cholesterol metabolism. Despite plausible mechanisms of its action, there is not enough scientific evidence to support the use of guggulipid for hyperlipidemia [85,89].

Nohr et al. researched the use of 2160 mg guggul daily or placebo on 43 women and men [90]. After 12 weeks, mean total cholesterol in the active group was significantly reduced compared with the placebo group β value with 95% CI: 0.346 (0.004 to 0.688, *p* = 0.047). However, the mean levels of LDL-C did not change significantly.

*Probiotic (Bifidobacterium longum* BB536). The intake of probiotics reduces total cholesterol and LDL in mildly hypercholesterolaemic subjects [91]. Probiotics strains such as *Bifidobacterium longum* BB536 show high biliary salt hydrolase activity [92]. *B. longum* BB536 contributes to lower circulating total cholesterol and LDL levels by reducing intestinal cholesterol reabsorption [93]. There are few possible mechanisms of action of probiotics on cholesterol removal. *B. longum* BB536 can incorporate cholesterol in its cell membranes leading to inhibition of intestinal cholesterol and decreasing serum levels [94]. The increased excretion of bile acids could also contribute to the lipid-lowering effect [95].

The single lipid-lowering effect of *B. longum* BB536 still needs to be investigated by RCT. However, a meta-analysis that researched the effects of other probiotics consumption on lipid profile and CVD risk factors demonstrates that probiotic use is effective in lipid-lowering [96].

*Catechin/theaflavins*. Polyphenols in tea include catechins, theaflavins, tannins, and flavonoids [97]. The mechanisms of action of tea flavonoids on cholesterol levels do not fully understand. Some results have suggested the inhibitory mechanism of intestinal cholesterol absorption by interfering with the formation of mixed micelles [98]. Catechin is a natural phenol. It has antioxidant properties and reduces LDL levels in humans [99].

A clinical trial studied 240 subjects on a low-fat diet with mild to moderate hypercholesterolemia the effect of daily use of 375 mg theaflavin-enriched green tea extract or placebo. After 12 weeks, the mean ± SEM changes from baseline in total cholesterol were −11.3 ± 0.9% (*p* = 0.01) and for LDL cholesterol −16.4 ± 1.1% (*p* = 0.01), respectively, in the tea extract group [100]. Another trial investigated the effects of a green tea supplement containing 1315 mg of catechins on serum lipids in postmenopausal women. After 12 months of administration, the results demonstrated a significant reduction in circulating total cholesterol in treated group compared with placebo (−2.1% vs. 0.7%; *p* = 0.0004) and LDL cholesterol (−4.1% vs. 0.9%; *p* < 0.0001) [101].

*Pine Bark Extract* (*Pinus Pinaster*). Pycnogenol is an extract of *Pinus pinaster*. The effect of this extract on oxidative stress and lipid profile in humans was studied. Pine bark extract increases plasma antioxidant capacity and HDL while it reduces LDL levels [102]. A clinical study analyzed 24 participants that received two capsules/day with 75 mg of oligopin, a quantified extract from French maritime pine bark with low molecular weight procyanidins, or placebo. At 5 weeks, compared to the placebo, oligopin raised high density lipoprotein cholesterol by 14% (*p* = 0.012), and decreased oxidized LDL concentrations by 31.72 U/l (*p* = 0.015) [103].

## 4. Study Design in the Selected Clinical Trials

RCTs are a gold standard for clinical research to verify the efficacy and safety of a novel NC [104]. RCTs study designs comprehend four major categories: parallel, crossover, cluster, and fractional [105]. All our selected studies to verify the lipid-lowering efficacy and safety of nutraceutical combinations were RCTs. The study design, the population description, and the lipid-lowering effects of NCs on cholesterol levels are reported in Table 1.

Among our selected studies, it appeared that twelve of fourteen studies followed the parallel design (Table 1). From these twelve studies with parallel design, particularly in the study of Trautwein EA et al. [19], three parallel groups of treatment (black tea theaflavins alone, black tea theaflavins in combination with catechin and placebo) were analyzed. Considering that only the theaflavins/catechin group vs. placebo met the topic of this review, we considered only the results of the NC vs. PL arm (Table 1). In the study of D′Addato S et al. [20], NC combination vs. PL was analyzed, while the data of active comparator was excluded (Table 1). In the study of Affuso F et al. [15] it was considered only NC data vs. PL after six weeks of treatment, while the subsequent open-label extension of 4 weeks, without placebo, was not of interest to this review, and it was not reported in Table 1. 

One among the selected studies was a crossover type, in which each patient received treatment and placebo but in a different order and timing [12]. The advantage of this design is the requirement of a smaller sample size compared to the parallel design, which often requires many patients. Only one from the selected sample study appeared to be 2 × 2 fractional type [16]. Each participant was randomly assigned to a group that receives a particular combination of interventions or non-interventions. With this type of design, one trial can answer two or more research questions [105]. In fact, in the reported fractional study [16], there were four treatment groups. We considered only NC vs. placebo arm, that meet our review topic.

## 5. Discussion

Nutraceuticals have the potential of reducing the incidence of various chronic degenerative diseases since they exert preventive and therapeutic activities [106,107]. NCs with different lipid-lowering mechanisms of action could prevent CVDs in patients with moderate hypercholesterolemia. The potential advantages of using NCs rather than a single nutraceutical compound arise from the idea of their possible additive or synergistic lipid-lowering effects.

Thousands of nutraceuticals are already discovered. An exponentially increasing number of NCs are becoming available on the market against hypercholesterolemia, leading to the research of the effect of their combinations.

However, in the literature, there is little evidence of their actual effects on humans. In particular, RCTs on the safety and efficacy of lipid-lowering NCs are scarce. In fact, despite an enormous number of NCs available in the market, we found only fourteen RCTs focused on the use of combined nutraceuticals, in the form of capsules, to lower the total cholesterol in hypercholesterolemic subjects.

In our RCTs sample, the study period range was from four [16,20] to twenty-four [14] weeks. Moreover, the subjects′ range starts from thirty participants in crossover study design [12] to one hundred and six subjects in another study with parallel design [20]. In all RCTs that have been investigated, populations consisted of both genders.

Regarding the efficacy of these mixtures, RCTs demonstrated that almost all NCs tested effectively lower lipid levels. In particular, 13 of 14 RCTs verified lipid-lowering effects while only one [19] reported no changes. These results support the possibility of using specific NCs as an alternative approach also in subjects that refuse drug therapy.

**Figure 1:**
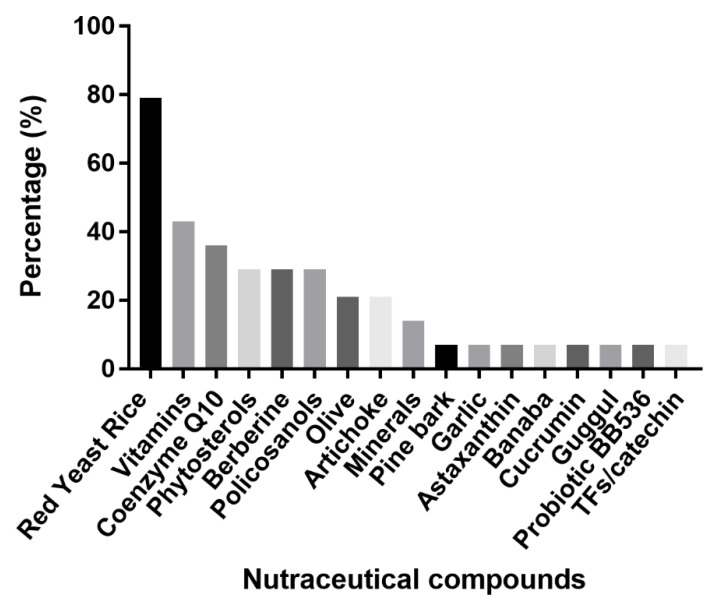
Percentage (%) of each compound present in NCs in the selected RCTs.

Interestingly, RYR has been used in the mixture of eleven among thirteen effective NCs (Figure 1).

Monacolin K, the lipid-lowering active compound of the RYR, exists in two forms, lactone and acidic [108]. Its lactone molecular form is identical to the hypocholesterolemic drug lovastatin. When ingested, both Monacolin K and lovastatin convert their molecular form to the bioactive hydroxyl acid form with inhibitory activity on HMG-CoA reductase. The difference between the drug and the active nutraceutical compound is that lovastatin as a prodrug needs to be converted from the lactone form in an acidic one. In contrast, an active acidic form already exists in RYR [108]. Literature data reported that Monacolin K from RYR taken from 3 to 11.4 mg daily reduces LDL levels from 14.8% to 26.3% [109,110]. In our selected RCTs, the sample used was RYR component Monakolin K from 3.33 mg to 10 mg, and the outcome was similar; nevertheless, other compounds were present in the NCs (Table 1). The question arising from these observations is whether the lipid-lowering efficacy of these combinations derives mainly from the monacolin K “natural statin” single effect or other compounds in the mixture. However, the importance of the other compounds in the mixture could also arise from their beneficial effects on different targets involved in the atherosclerotic process. As discussed above in detail, all these compounds exert preventive actions against different mechanisms such as oxidative stress, inflammation, and lipid metabolism.

Up to now, few studies have verified the efficacy of every single compound in the mixture vs. NCs to evaluate possible additive or synergistic effects. Probably for the time and high resources request depending on the number of nutraceutical candidates and their possible combinations to perform the studies [106]. However, it will be more feasible to investigate the effect of one single candidate of interest vs. NCs. For example, it will be interesting to explore the different lipid-lowering effectiveness of RYR vs. NCs with RYR. It could be possible to use 2 × 2 fractional intervention in four parallel groups: NC combination with RYR, the same NC combination without RYR, single RYR, and placebo. Considering that all NC RYRs are effective in lipid lowering, it would be important to further understand the roles of other compounds in each RYR combination with targeted analyses.

Scientific opinions of the safety of the monacolin K have been put forward [26]. It is reported that the adverse effects of the natural monacolin K are similar to those of synthetic statins. RYR is tolerated in lower doses (3 mg/day) up to a high dose (10 mg/day) of monacolin K [111]. However, adverse effects of RYR are symptomatic myopathy, gastrointestinal symptoms, and elevated levels of hepatic enzymes [112,113]. Among our selected papers, one article [20] reported insomnia as a treatment-related adverse effect of NC containing RYR. It seems like there is an underestimation of adverse events from RYR since the nutraceutical compounds are natural products but not drugs. As a consequence, they are usually perceived as “safe” and without side effects. In fact, EFSA Panel on Food Additives and Nutrient Sources added to Food (ANS) was not identified a dietary intake of monacolins from RYR that does not give rise to concerns of its harmful effects to the general population health and vulnerable subgroups 26.

However, it is relevant for the RYR intolerant populations to have the possibility to use NCs without the presence of Monacolin-K. Our sample contained three NCs that did not include RYR [16,19,23]. Two of these three combinations showed lipid-lowering efficacy [16,23].

In conclusion, to manage the arising nutraceutical tide, we strongly suggest increasing the number of clinical studies to evaluate the efficacy and safety of the new NCs. Moreover, in our opinion, robust design such as RCTs should be the preferred choice when planning a clinical trial on NCs in hypercholesterolemia. In addition, to verify possible additive and/or synergistic effects, the new nutraceutical candidates should be individually tested by their use in additional arms of subjects vs. NCs and placebo arms in the RCTs.

This review offers a picture of the main scientific RCTs evidence to evaluate NCs as an alternative approach also in subjects that refuse lipid-lowering drug therapy.

## Figures and Tables

**Table 1:** The nutraceutical combinations with the relative concentration of single compounds and description of research methods and effectiveness of the nutraceutical combinations in the selected RCTs.

Refs	Natural Active Components	Type of RCT	Study Intervention Period (Weeks)	Total Subjects (*n*)	Male (*n*)	Female (*n*)	Age Range (Years)	LDL-Cholesterol (LDL-C)					Total Cholesterol (TC)					Adverse Effects
								Intervention Group		Placebo Group		Difference of Changes between Arms	Intervention Group		Placebo Group		Difference of Changes between Arms	
								Baseline vs. Final Mean LDL-C ± SD, (mg/dL)	LDL-C Reduction	Baseline vs.Final Mean LDL-C ± SD (mg/dL)	LDL-C Reduction	*p* Value	Baseline vs. Final Mean TC ± SD (mg/dL)	TC Reduction	Baseline vs. Final Mean TC ± SD (mg/dL)	TC Reduction	*p* Value	
[10]	*Bifidobacterium longum* BB536 (1 bn UFC) Coenzyme Q10 (20 mg) Niacin (16 mg) RYR extract (10 mg monacolin K)	parallel	12	33	16	17	18–70	177 (167, 193) ^1^ vs. 136.5 (118,151.5) ^1^	−25.7%	189 (174, 198) ^1^ vs. 183 (171, 202) ^1^	NA	<0.0001	271 (239, 285) ^1^ vs. 208 (201, 263) ^1^	−16.7%	271 (256, 289) ^1^ vs. 267 (259, 293)	NA	<0.0001	No
[11]	Phytosterols 1.5 g Vitamin E (12 mg) RYR extract (10 mg monacolin K) Olive leaf extract (5 mg hydroxytyrosol)	parallel	12	40	13	27	35–75	150.1 (142, 159.8) ^1^ vs. 120 (112.7, 129.3) ^1^	−19.7%	143.2 (133.9, 152.1) ^1^ vs. 138.6 (130.5, 145.9) ^1^	−6.7%	<0.001	234.3 (225.1, 244.0) ^1^ vs. 201.5 (215.4, 234.7) ^1^	−13.9%	225.1 (215.4, 234.7) ^1^ vs. 218.5 (207.7, 229.3) ^1^	−3.0%	<0.001	No
[12]	Artichoke extract (500 mg) Banaba extract (75 mg) Coenzyme Q10 (50 mg) RYR 200 (10 mg monacolin K) Vitamin B3 (9 mg) Vitamin B6 (1.4 mg) Vitamin B12 (0.83 mcg) Folic acid (110 mcg)	crossover	6-2-6	30	NA	NA	25–75	NA	−30.3 mg/dL	NA	−8.4 mg/dL	<0.05	NA	−34.1 mg/dL	NA	−10.1 mg/dL	<0.05	No
[13]	Phytosterols (400 mg) RYR extract (5 mg monacolin K) L-tyrosol (2.5 mg)	parallel	8	50	30	20	35–69	162.3 ± 21.9 vs. NA	−38.0 mg/dL	151.7 ± 24.3 vs. NA	−20 mg/dL	<0.05	234.5 ± 24.7 vs. NA	−38.2 mg/dL	225.1 ± 25.3 vs. NA	−22.2 mg/dL	<0.05	No
[14]	Coenzyme Q10 (30 mg) RYR extract (10 mg monacolin K)	parallel	24	40	18	22	18–70	158.5 ± 15.4 vs. 116.8 ± 9.9	−26.3%	161.6 ± 13.8 vs. 167.1 ± 14.2	+3.4%	<0.05	229.1 ± 14.1 vs. 180.7 ± 15.3	−21.1%	233.4 ± 12.8 vs. 238.4 ± 14.6	+2.1%	<0.05	No
[15]	Berberine (500 mg) RYR extract (10 mg monacolin K) Policosanols (10 mg)	parallel	6	50	26	24	18–70	175.6 ± 25.1 vs. NA	−40.9 mg/dL	170.6 ± 22.0 vs. NA	−1.5 mg/dL	<0.001	254.8 ± 28.9 vs. NA	−44.0 mg/dL	250.9 ± 30.8 vs. NA	−1.16 mg/dL	<0.001	No
[16]	Phytosterols (2 g) Curcumin (200 mg)	2 × 2 fractional	4	37 ^2^	NA	NA	18–70	166.8 ± 5.8 ^3^ vs. 142.4 ± 6.2 ^3^	−14.42%	175.6 ± 6.9 ^3^ vs. 172.9 ± 7.3 ^3^	−0.89%	0.006	251.3 ± 7.3 ^3^ vs. 222.3 ± 6.2 ^3^	−11.01%	255.9 ± 6.9 ^3^ vs. 252.9 ± 8.1 ^3^	−1.23%	0.005	No
[17]	Berberine (500 mg) RYR 200 mg (10 mg monacolin K) Policosanol (10 mg) Coenzyme Q10 (2 mg) Astaxanthin (0.5 mg) Folic acid (0.2 mg)	parallel	12	104	32	72	˃18	155.7 ± 14.6 vs. 135.3 ± 28.7	−14.93%	159.3 ± 15.6 vs. 147.5 ± 26.0	−8.02%	0.029	243.6 ± 24.3 vs. 221.8 ± 34.4	−10.46%	243.4 ± 19.5 vs. 232.8 ± 28.8	−5.5%	0.01	No
[18]	Artichoke leaf dry extract (200 mg) RYR 166.67 mg (0.4% monacolin K) Dicalcium phosphate (199 mg) Microcrystalline cellulose (87.36 mg) Calcium citrate (63.22 mg) Tricalcium phosphate (34 mg) Magnesium stearate (22 mg) Vitamin E (12.86 mg) Garlic dry extract (10 mg) Pine bark extract (6.67 mg) Sugar cane extract (3.70 mg) Vitamin B2 (1.60 mg) Vitamin B3 (2.92 mg)	parallel	16	39	11	28	21–55	170.0 ± 20.0 vs. NA	−19.1% *p* < 0.001 vs. baseline	170.0 ± 30.0 vs. NA	+2.8% *p* = 0.37 vs. baseline	NA	250.0 ± 30.0 vs. NA	−14.1% *p* < 0.001 vs. baseline	250.0 ± 30.0 vs. NA	+1.3 *p* = 0.53 vs. baseline	NA	No
[19]	Catechins (149.4 mg) Purified black tea theaflavins (75 mg)	parallel	11	66 ^2^	44	22	18–65	150.5 ± 25.8 vs. 151.3 ± 27.4	NA	154.0 ± 18.9 vs. 151.3 ± 17.8	NA	0.106	223.1 ± 32.0 vs. 221.6 ± 32.8	NA	217.3 ± 21.6 vs. 215.8 ± 21.62	NA	0.118	Yes
[20]	Berberine (500 mg) RYR (10 mg monacolin K) Hydroxytyrosol (5 mg) Coenzyme Q10 (2 mg)	parallel	4	106 ^2^	NA	NA	18–75	147.5 ± 16.3 vs. 120.4 ± 18.8	−39.1 mg/dL	143.6 ± 15.0 vs. 149.3 ± 19.5	+5.7 mg/dL	<0.001	234.6 ± 18.0 vs. 204.9 ± 22.2	−45.9 mg/dL	235.6 ± 17.9 vs. 238.0 ± 21.5	+2.4 mg/dL	<0.001	Yes
[21]	Phytosterols (800 mg) Niacin (27 mg) Policosanols (10 mg) RYR extract (5 mg monacolin K)	parallel	8	88	38	47	30–75	155.1 ± 19.9 vs. 122.6 ± 24.7	−32.5 mg/dL	161.5 ± 21.3 vs. 164.0 ± 20.4	+2.5 mg/dL	<0.01	229.1 ± 27.9 vs. 197.1 ± 28.3	−33.0 mg/dL	232.6 ± 21.6 vs. 239.8 ± 23.6	+8.2 mg/dL	<0.01	No
[22]	RYR extract 375 mg (3.75 mg monacolin K) Guggul extract (110 mg) Chromium picolinate (50 μg)	parallel	8	80	38	42	18–65	137.6 ± 20.2 vs. 115.5 ± 22.2	−16.1%	134.4 ± 20.5 vs. 145.1 ± 23.7	+8.0%	<0.05	205.0 ± 17.6 vs. 173.5 ± 21.7	−15.4%	200.8 ± 22.9 vs. 204.5 ± 22.8	+1.8%	<0.05	Yes
[23]	Artichoke leaf extract ^4^ Berberine ^4^	parallel	8	40	NA	NA	˃18	156.7 ± 10.9 vs. 131.9 ± 9.1	−16%	157.4 ± 9.8 vs. 136.7 ± 10.8	NA	<0.01	247.1 ± 11.7 vs. 199.9 ± 10.2	−19.0%	245.8 ± 13.6 vs. 223.6 ± 11.9	NA	<0.01	No

NA, not available; TC, total cholesterol; LDL-C, LDL cholesterol; ^1^ median (interquartile range); ^2^ nutraceutical combination and placebo group; ^3^ means ± SEM; ^4^ concentration not reported. To be consistent with the units of studies presented in the table, when appropriate, we recalculated reduction of TC and LDL-C in mg/dL.
